# Supplementary material for: Relationships between light exposure and aspects of cognitive function in everyday life
Source: Commun Psychol. 2025 Dec 16;4:5. doi: 10.1038/s44271-025-00373-9 (PMC12789024; doi:10.1038/s44271-025-00373-9)
Supplement: Supplementary file 2 — Supplementary information [file 44271_2025_373_MOESM2_ESM.pdf]

Supplementary information

Figure S1: The learning effect was investigated for subjective sleepiness reports (KSS), PVT reaction time and accuracy, NB3 false positive rate (FPR) and false negative rate (FNR), as well as visual search (VS) inverse efficiency score (IES), FPR, and FNR. A linear mixed model with random participant intercepts was used to estimate changes in cognitive variables across task days. This model also controlled for interaction effects between the number of games played daily and task day. KSS and PVT showed no significant learning effect and were not influenced by the number of tasks completed per day. In contrast, NB3 demonstrated significant improvement across task days. Specifically, NB3 median reaction time decreased by 16.8 ms per task day ($p = 0.008$), FPR decreased by 1.5% per task day ($p = 1.85e-06$), and FNR decreased by 1.6% per task day ($p = 0.006$). VS also showed significant improvement across task days, with the IES decreasing by 241.2 ms/% per task day ($p < 2e-16$). Additionally, VS was influenced by the number of tasks completed per day, with each additional session played reducing the IES by 40.2 ($p = 0.023$). Overall, PVT was independent of the number of tasks completed per day and showed no learning effect. In contrast, NB3 and VS, which were slightly more complex tasks, exhibited a learning effect. Increased task repetition led to greater practice effects, though this was only observed in VS IES.

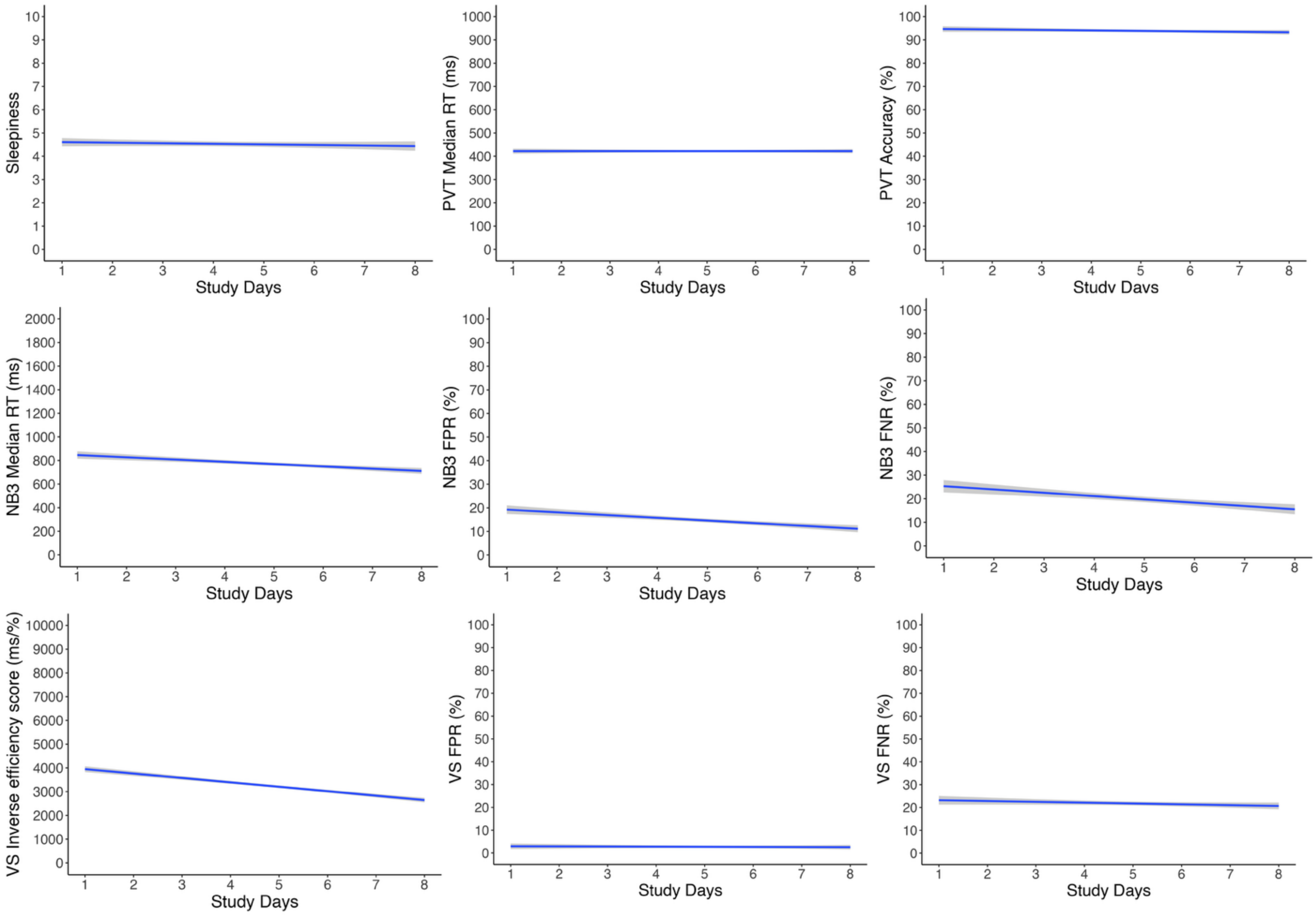

Supplementary information

Figure S2: Brightertime app entries per participant.

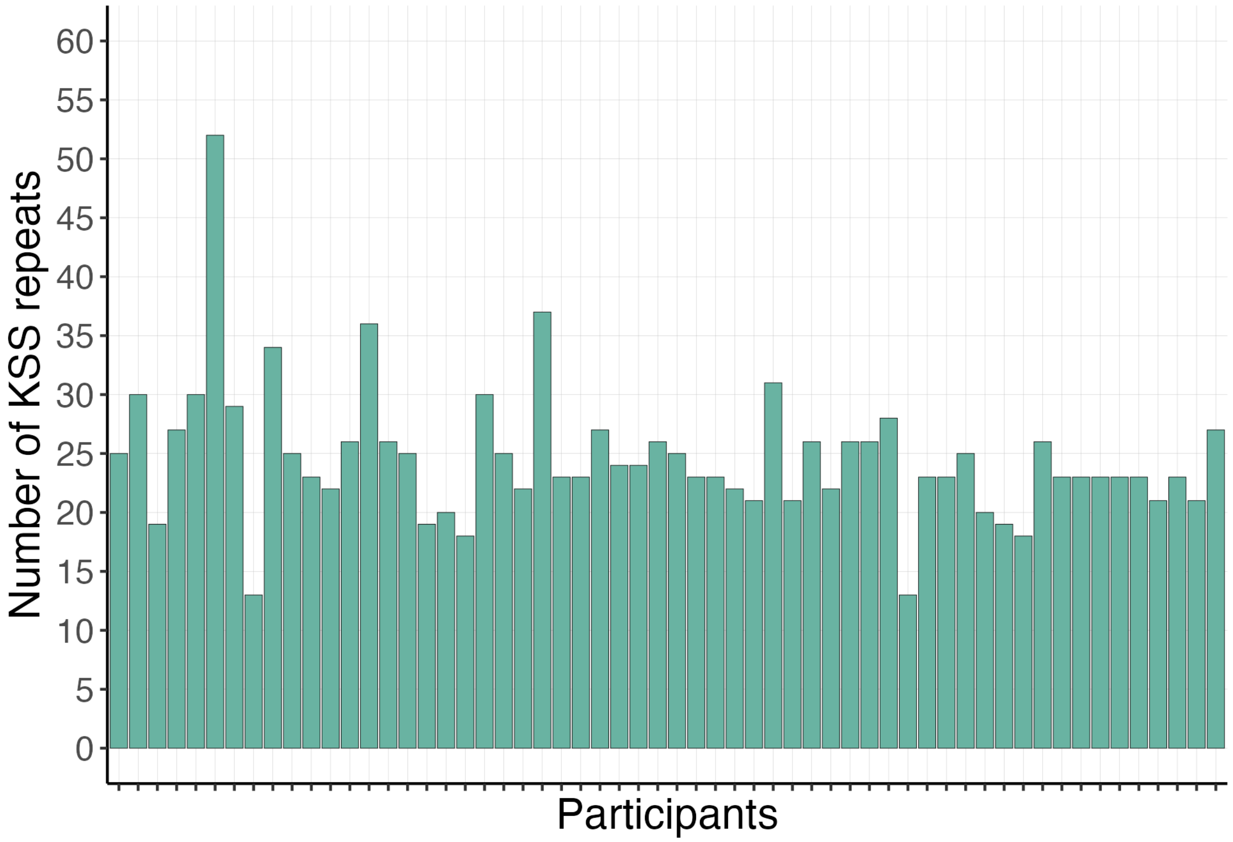

Supplementary information

Figure S3: Study design (representative data structure for one participant). Lines show a sinusoidal fit with a 24-hour period. Abbreviations: Mel EDI – Melanopic Daylight Illuminance; KSS – Karolinska Sleepiness Scale.

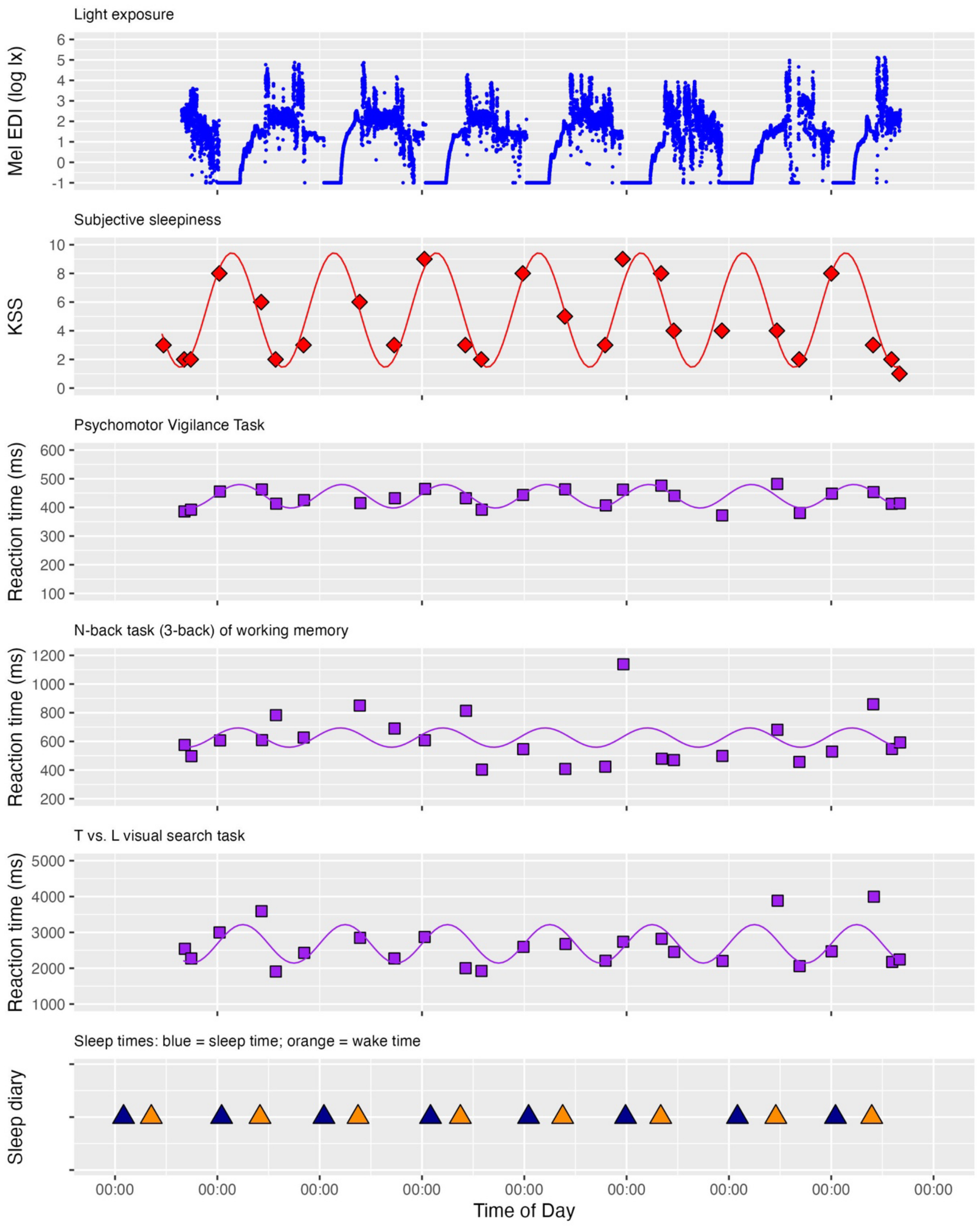

Supplementary information

Figure S4: Distributions of cognitive variables with the maximum factor loadings.

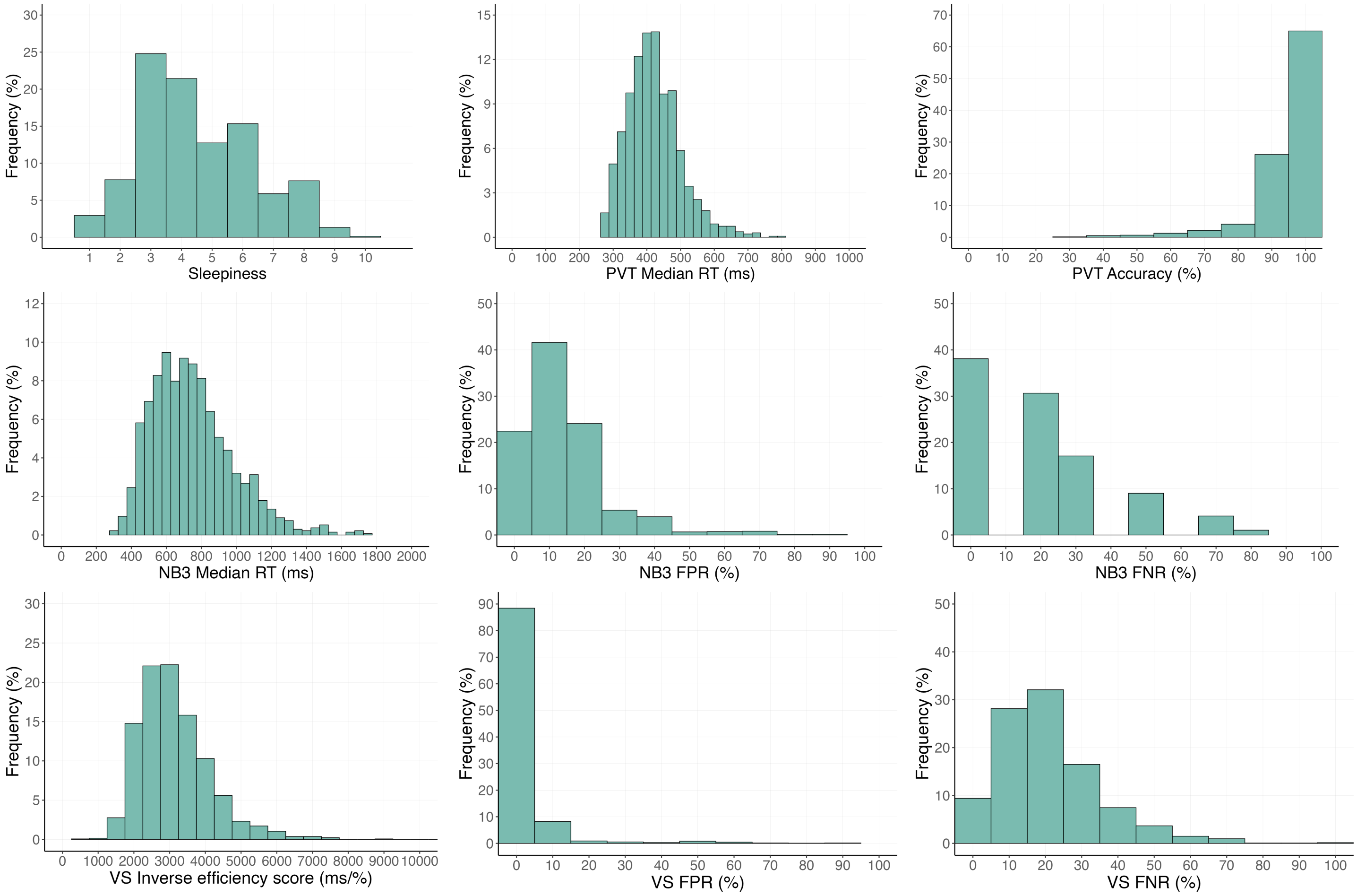

Supplementary information

Figure S5: Scatterplots highlighting key findings Figure 4. **A)** Midpoint of the dimmest 5-hour period vs. KSS melanopic photosensitivity slope. **B)** M10 (Mel EDI lux) vs. VS false positive rate (%), based on weekly averages. **C)** Subjective brightness from in-lab pupillometric assay vs. VS false negative rate (%).

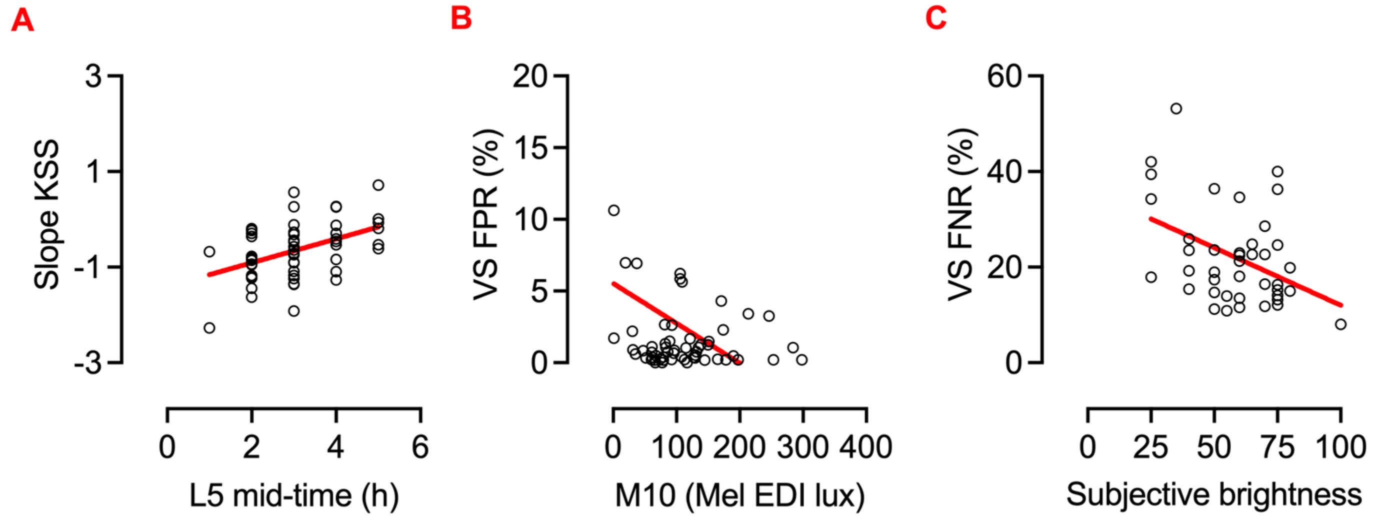

Supplementary information

Table S1: Summary statistics for all cognitive outcome variables.

	MIN.	1ST QU.	MEDIAN	MEAN	3RD QU.	MAX.	STD.DEV
							**Psychomotor Vigilance Task (PVT)**
**FNR**	0.000	0.000	0.000	3.080	3.571	62.963	6.656
**FDR**	0.000	0.000	0.000	3.414	3.704	65.385	6.776
**ACCURACY**	32.143	92.857	96.429	93.899	100.000	100.000	9.370
**MEDIANRT**	263.000	369.000	413.750	421.531	470.000	800.000	79.309
**IES**	274.964	397.088	453.929	483.272	523.767	1869.560	153.160
**SLOW10**	289.000	460.400	533.700	551.455	621.750	953.400	128.418
**FAST10**	236.200	313.600	350.600	354.147	386.675	682.600	58.617
**LAPSE**	0.000	2.000	4.000	6.115	9.000	27.000	5.807
							**N-Back Task (3-back)**
**FNR**	0.000	0.000	16.667	18.916	33.333	83.333	19.943
**FPR**	0.000	6.250	12.500	13.754	18.750	93.750	13.549
**FDR**	0.000	14.286	25.000	27.583	42.857	85.714	21.025
**FOR**	0.000	0.000	6.250	7.537	12.500	42.857	7.859
**ACCURACY**	31.818	77.273	86.364	84.838	95.455	100.000	12.620
**DPRIME**	-0.629	1.249	2.094	1.974	2.681	3.355	0.926
**MEDIANRT**	285.500	577.000	719.000	750.481	878.000	1755.000	234.328
**IES**	337.167	716.152	909.975	961.841	1141.486	2899.769	343.581
**SLOW10**	360.000	798.900	996.500	1028.424	1229.800	1888.000	307.159
**FAST10**	115.000	455.000	540.200	581.899	659.000	1755.000	183.078
							**Visual search (VS)**
**FNR**	0.000	11.111	19.139	21.318	28.571	100.000	14.128
**FPR**	0.000	0.000	0.000	2.474	0.000	86.957	8.656
**FDR**	0.000	0.000	0.000	2.927	0.000	70.588	9.113
**FOR**	0.000	9.091	16.000	17.352	23.077	70.000	10.620
**ACCURACY**	38.095	85.714	90.476	87.921	95.238	100.000	9.563
**DPRIME**	-0.632	2.385	2.718	2.663	3.019	4.001	0.698
**MEDIANRT**	295.000	2088.375	2541.750	2637.490	3076.125	7523.500	821.914
**IES**	575.423	2431.322	2959.385	3134.033	3640.271	15273.615	1043.709
**SLOW10**	455.500	3258.650	3975.600	4210.635	4991.025	9852.000	1325.897
**FAST10**	206.000	1035.175	1225.000	1300.882	1494.900	4826.400	425.862
**SLOPE**	-1973.975	399.432	694.819	746.680	1049.531	2887.974	513.565

Supplementary information

Table S2: Correlation matrices and factor loadings of cognitive variables.

	FNR	FDR	ACC	MEDIANRT	IES	SLOW10	FAST10	LAPSE	FPR	FOR	DPRIME	SLOPE
									**Psychomotor Vigilance Task (PVT) - Correlation matrix**			
**FNR**	1.000	0.322	-0.808	0.463	0.782	0.519	0.326	0.302				
**FDR**	0.322	1.000	-0.803	0.122	0.587	0.188	0.029	0.002				
**ACCURACY**	-0.808	-0.803	1.000	-0.370	-0.831	-0.450	-0.226	-0.202				
**MEDIANRT**	0.463	0.122	-0.370	1.000	0.766	0.858	0.912	0.888				
**IES**	0.782	0.587	-0.831	0.766	1.000	0.770	0.634	0.595				
**SLOW10**	0.519	0.188	-0.450	0.858	0.770	1.000	0.736	0.824				
**FAST10**	0.326	0.029	-0.226	0.912	0.634	0.736	1.000	0.839				
**LAPSE**	0.302	0.002	-0.202	0.888	0.595	0.824	0.839	1.000				
										**N-Back Task (3-back) - Correlation matrix**		
**FNR**	1.000	0.597	-0.668	0.197	0.445	0.013	0.298		0.304	0.963	-0.822	
**FPR**	0.304	0.882	-0.912	0.114	0.578	0.081	0.105		1.000	0.476	-0.757	
**FDR**	0.597	1.000	-0.946	0.193	0.606	0.099	0.222		0.882	0.696	-0.921	
**FOR**	0.963	0.696	-0.787	0.193	0.531	0.025	0.278		0.476	1.000	-0.886	
**ACCURACY**	-0.668	-0.946	1.000	-0.174	-0.643	-0.069	-0.211		-0.912	-0.787	0.945	
**DPRIME**	-0.822	-0.921	0.945	-0.222	-0.616	-0.098	-0.266		-0.757	-0.886	1.000	
**MEDIANRT**	0.197	0.193	-0.174	1.000	0.791	0.770	0.835		0.114	0.193	-0.222	
**IES**	0.445	0.606	-0.643	0.791	1.000	0.707	0.746		0.578	0.531	-0.616	
**SLOW10**	0.013	0.099	-0.069	0.770	0.707	1.000	0.561		0.081	0.025	-0.098	
**FAST10**	0.298	0.222	-0.211	0.835	0.746	0.561	1.000		0.105	0.278	-0.266	
										**Visual search (VS) - Correlation matrix**		
**FNR**	1.000	0.393	-0.860	-0.059	0.281	-0.051	0.070		0.273	0.864	-0.833	-0.101
**FPR**	0.273	0.924	-0.664	-0.257	0.031	-0.136	-0.239		1.000	0.444	-0.698	-0.204
**FDR**	0.393	1.000	-0.710	-0.230	0.084	-0.125	-0.196		0.924	0.452	-0.757	-0.187
**FOR**	0.864	0.452	-0.907	-0.142	0.224	-0.083	-0.068		0.444	1.000	-0.861	-0.143
**ACCURACY**	-0.860	-0.710	1.000	0.146	-0.282	0.067	0.055		-0.664	-0.907	0.948	0.166
**DPRIME**	-0.833	-0.757	0.948	0.158	-0.227	0.090	0.066		-0.698	-0.861	1.000	0.160
**MEDIANRT**	-0.059	-0.230	0.146	1.000	0.861	0.896	0.800		-0.257	-0.142	0.158	0.571
**IES**	0.281	0.084	-0.282	0.861	1.000	0.873	0.766		0.031	0.224	-0.227	0.451
**SLOW10**	-0.051	-0.125	0.067	0.896	0.873	1.000	0.697		-0.136	-0.083	0.090	0.558
**FAST10**	0.070	-0.196	0.055	0.800	0.766	0.697	1.000		-0.239	-0.068	0.066	0.392
**SLOPE**	-0.101	-0.187	0.166	0.571	0.451	0.558	0.392		-0.204	-0.143	0.160	1.000

Supplementary information

	FACTOR1	FACTOR2	FACTOR3
			**Psychomotor Vigilance Task (PVT) - Loading matrix**
**FNR**	0.311	0.767	
**FDR**	-0.057	0.822	
**ACCURACY**	-0.163	-0.984	
**MEDIANRT**	0.967	0.216	
**IES**	0.626	0.742	
**SLOW10**	0.819	0.322	
**FAST10**	0.923	0.077	
**LAPSE**	0.907	0.055	
			**N-Back Task (3-back) - Loading matrix**
**FNR**	0.218	0.111	0.967
**FPR**	0.992	0.074	0.083
**FDR**	0.847	0.097	0.415
**FOR**	0.395	0.130	0.891
**ACCURACY**	-0.868	-0.106	-0.482
**DPRIME**	-0.697	-0.119	-0.678
**MEDIANRT**	0.041	0.907	0.090
**IES**	0.500	0.825	0.253
**SLOW10**	0.027	0.865	-0.091
**FAST10**	0.028	0.823	0.206
			**Visual search (VS) - Loading matrix**
**FNR**	0.015	0.961	0.039
**FPR**	-0.135	0.248	0.957
**FDR**	-0.102	0.354	0.860
**FOR**	-0.049	0.909	0.220
**ACCURACY**	0.008	-0.878	-0.465
**DPRIME**	0.047	-0.815	-0.511
**MEDIANRT**	0.945	-0.090	-0.113
**IES**	0.950	0.278	0.094
**SLOW10**	0.937	-0.068	0.007
**FAST10**	0.808	0.031	-0.145
**SLOPE**	0.531	-0.124	-0.106

Supplementary information

Table S3: Mixed model results for cognitive measures in relation to sleep duration, sleep midpoint, time awake, and time of day (standardized coefficient, p value, sample size, effect size).

	KSS	PVT MEDIANRT	PVT ACCURACY	NB3 MEDIANRT	NB3 FPR	NB3 FNR	VS IES	VS FPR	VS FNR
									**Model coefficients**
**SIN (TIME OF DAY)**	0.237	0.021	0.029	0.055	-0.024	-0.026	0.002	-0.007	-0.036
**COS (TIME OF DAY)**	0.441	0.074	-0.039	-0.012	0.029	0.022	0.029	0.029	0.059
**TIMEAWAKE**	-1.042	-0.099	-0.091	-0.212	0.039	0.124	-0.113	0.026	0.111
**TIMEAWAKE^2**	1.361	0.183	0.051	0.186	0.014	-0.077	0.154	0.001	0.001
**SLEEP DURATION**	-0.096	-0.085	0.031	-0.029	0.000	-0.022	-0.039	0.017	-0.071
**SLEEP MIDPOINT**	0.024	-0.047	0.010	-0.036	-0.081	-0.041	-0.106	-0.007	-0.099
									**Model p-values**
**SIN (TIME OF DAY)**	7.85E-09	2.39E-01	1.74E-01	2.65E-02	2.78E-01	3.03E-01	9.10E-01	7.75E-01	1.79E-01
**COS (TIME OF DAY)**	1.96E-14	4.71E-03	1.84E-01	6.60E-01	2.14E-01	3.87E-01	1.71E-01	1.90E-01	4.38E-02
**TIMEAWAKE**	5.13E-09	5.39E-01	2.27E-01	2.75E-01	8.12E-01	5.47E-01	5.14E-01	7.67E-01	3.03E-01
**TIMEAWAKE^2**	3.29E-12	2.84E-01	5.33E-01	3.25E-01	9.30E-01	7.08E-01	3.65E-01	9.93E-01	9.95E-01
**SLEEP DURATION**	2.96E-03	2.57E-04	2.53E-01	3.64E-01	9.99E-01	4.95E-01	1.13E-01	4.88E-01	4.39E-02
**SLEEP MIDPOINT**	5.83E-01	2.22E-01	7.56E-01	3.39E-01	8.94E-02	3.03E-01	3.73E-02	8.27E-01	6.09E-03
									**Model sample sizes**
**SIN (TIME OF DAY)**	1405.000	1312.000	1312.000	1323.000	1323.000	1323.000	1319.000	1319.000	1319.000
**COS (TIME OF DAY)**	1405.000	1312.000	1312.000	1323.000	1323.000	1323.000	1319.000	1319.000	1319.000
**TIMEAWAKE**	1405.000	1312.000	1312.000	1323.000	1323.000	1323.000	1319.000	1319.000	1319.000
**TIMEAWAKE^2**	1405.000	1312.000	1312.000	1323.000	1323.000	1323.000	1319.000	1319.000	1319.000
**SLEEP DURATION**	1405.000	1312.000	1312.000	1323.000	1323.000	1323.000	1319.000	1319.000	1319.000
**SLEEP MIDPOINT**	1405.000	1312.000	1312.000	1323.000	1323.000	1323.000	1319.000	1319.000	1319.000
									**Model effect sizes (eta-squared)**
**SIN (TIME OF DAY)**	0.476	0.002	0.023	0.029	0.004	0.002	0.000	0.002	0.030
**COS (TIME OF DAY)**	0.673	0.143	0.034	0.003	0.033	0.001	0.002	0.022	0.088
**TIMEAWAKE**	0.517	0.002	0.012	0.037	0.000	0.003	0.007	0.001	0.019
**TIMEAWAKE^2**	0.643	0.009	0.006	0.034	0.000	0.001	0.015	0.000	0.000
**SLEEP DURATION**	0.306	0.079	0.020	0.027	0.000	0.014	0.004	0.006	0.137
**SLEEP MIDPOINT**	0.010	0.039	0.001	0.001	0.055	0.053	0.096	0.000	0.007

Supplementary information

Table S4: Mixed model results for recent melanopic EDI and cognitive measures (standardized coefficient, p value).

	KSS	PVT MEDIANRT	PVT ACCURACY	NB3 MEDIANRT	NB3 FPR	NB3 FNR	VS IES	VS FPR	VS FNR
									**Model coefficients**
**30-MIN**	-0.111	-0.085	0.001	-0.070	-0.046	-0.072	-0.026	0.006	0.004
**60-MIN**	-0.104	-0.056	-0.022	-0.083	-0.033	-0.053	-0.004	0.022	-0.024
**90-MIN**	-0.114	-0.047	-0.014	-0.084	-0.025	-0.041	0.012	-0.005	-0.010
**120-MIN**	-0.119	-0.029	-0.034	-0.078	-0.031	-0.037	0.016	-0.026	-0.012
									**Model p-values**
**30-MIN**	2.85E-03	3.49E-03	9.64E-01	3.35E-02	1.30E-01	3.57E-02	3.93E-01	8.67E-01	9.06E-01
**60-MIN**	1.26E-02	8.66E-02	4.69E-01	1.83E-02	3.41E-01	1.49E-01	8.98E-01	6.07E-01	5.10E-01
**90-MIN**	6.68E-03	1.27E-01	6.56E-01	2.50E-02	4.80E-01	2.71E-01	7.22E-01	8.58E-01	7.94E-01
**120-MIN**	4.65E-03	3.15E-01	2.49E-01	5.16E-02	4.53E-01	3.55E-01	6.42E-01	3.82E-01	7.56E-01

Supplementary information

Table S5: Correlations between weekly melanopic EDI exposure variables and real-world light sensitivity in cognitive measures (Pearson correlation r, p value, sample size).

	KSS SLOPE	PVT SLOPE	NB3 SLOPE
			**Pearson correlation r**
**M10**	-0.379	0.188	-0.140
**L5**	0.176	0.038	-0.071
**DURATION ABOVE 250 LUX**	-0.297	0.108	-0.281
**DURATION ABOVE 10 LUX AFTER SUNSET**	0.136	0.064	0.044
**M10 MID-TIME**	-0.036	-0.116	-0.086
**L5 MID-TIME**	0.443	-0.182	-0.078
**LAST TIME ABOVE 1 LUX**	0.307	-0.081	0.041
**IS**	-0.348	-0.045	0.086
**IV**	0.272	0.116	0.077
			**p-values**
**M10**	4.73E-03	1.69E-01	3.13E-01
**L5**	2.02E-01	7.84E-01	6.10E-01
**DURATION ABOVE 250 LUX**	2.89E-02	4.34E-01	3.95E-02
**DURATION ABOVE 10 LUX AFTER SUNSET**	3.26E-01	6.40E-01	7.51E-01
**M10 MID-TIME**	7.94E-01	3.99E-01	5.35E-01
**L5 MID-TIME**	7.89E-04	1.84E-01	5.77E-01
**LAST TIME ABOVE 1 LUX**	2.41E-02	5.58E-01	7.70E-01
**IS**	1.00E-02	7.44E-01	5.35E-01
**IV**	4.70E-02	3.99E-01	5.79E-01
			**sample sizes**
**M10**	54.000	55.000	54.000
**L5**	54.000	55.000	54.000
**DURATION ABOVE 250 LUX**	54.000	55.000	54.000
**DURATION ABOVE 10 LUX AFTER SUNSET**	54.000	55.000	54.000
**M10 MID-TIME**	54.000	55.000	54.000
**L5 MID-TIME**	54.000	55.000	54.000
**LAST TIME ABOVE 1 LUX**	54.000	55.000	54.000
**IS**	54.000	55.000	54.000
**IV**	54.000	55.000	54.000

Supplementary information

Table S6: Correlations between in-lab light sensitivity measures and real-world light sensitivity in cognition measures (Pearson correlation r, p value, sample size).

	KSS SLOPE	PVT SLOPE	NB3 SLOPE
			**Pearson correlation r**
**BRIGHTNESS .75 THRESHOLD**	0.197	0.244	0.153
**BRIGHTNESS PREFERENCE**	-0.113	0.215	-0.190
**PIPR RESPONSE**	0.341	-0.122	0.303
**PIPR RESPONSE (NORM)**	0.221	-0.123	0.213
**INITIAL CONSTRICTION**	-0.246	-0.332	-0.335
**SUBJECTIVE BRIGHTNESS**	0.086	0.019	-0.007
			**p-values**
**BRIGHTNESS .75 THRESHOLD**	3.44E-01	2.40E-01	4.76E-01
**BRIGHTNESS PREFERENCE**	4.98E-01	1.89E-01	2.52E-01
**PIPR RESPONSE**	3.35E-02	4.53E-01	6.09E-02
**PIPR RESPONSE (NORM)**	1.76E-01	4.49E-01	1.93E-01
**INITIAL CONSTRICTION**	1.30E-01	3.63E-02	3.71E-02
**SUBJECTIVE BRIGHTNESS**	6.03E-01	9.10E-01	9.68E-01
			**sample sizes**
**BRIGHTNESS .75 THRESHOLD**	25.000	25.000	24.000
**BRIGHTNESS PREFERENCE**	38.000	39.000	38.000
**PIPR RESPONSE**	39.000	40.000	39.000
**PIPR RESPONSE (NORM)**	39.000	40.000	39.000
**INITIAL CONSTRICTION**	39.000	40.000	39.000
**SUBJECTIVE BRIGHTNESS**	39.000	40.000	39.000

Supplementary information

Table S7: Correlations between weekly melanopic EDI exposure variables and average cognitive performance (Pearson correlation r, p value, sample size).

	KSS	PVT MEDIANRT	PVT ACCURACY	NB3 MEDIANRT	NB3 FPR	NB3 FNR	VS IES	VS FPR	VS FNR
									**Pearson correlation r**
**M10**	0.060	-0.325	0.222	0.054	-0.272	0.142	0.072	-0.527	-0.091
**L5**	-0.113	0.249	-0.183	0.081	0.143	0.109	0.134	0.144	0.129
**DURATION ABOVE 250 LUX**	0.147	-0.100	0.020	0.008	-0.032	0.242	0.091	-0.321	-0.009
**DURATION ABOVE 10 LUX AFTER SUNSET**	-0.091	0.159	0.106	0.040	0.025	0.080	-0.081	0.115	-0.049
**M10 MID-TIME**	-0.113	0.004	-0.110	-0.064	0.038	0.147	0.249	0.007	0.087
**L5 MID-TIME**	-0.053	0.333	-0.134	-0.080	0.223	-0.045	-0.138	0.235	0.167
**LAST TIME ABOVE 1 LUX**	-0.336	0.309	-0.081	0.084	0.184	0.091	0.143	0.231	0.134
**IS**	0.026	-0.322	0.302	0.134	-0.218	-0.099	-0.050	-0.275	-0.144
**IV**	0.052	0.332	-0.231	0.012	0.308	0.097	-0.042	0.436	0.087
									**p-values**
**M10**	6.63E-01	1.46E-02	1.01E-01	6.91E-01	4.26E-02	2.96E-01	5.96E-01	3.04E-05	5.06E-01
**L5**	4.08E-01	6.47E-02	1.76E-01	5.51E-01	2.91E-01	4.26E-01	3.24E-01	2.89E-01	3.42E-01
**DURATION ABOVE 250 LUX**	2.79E-01	4.64E-01	8.82E-01	9.53E-01	8.16E-01	7.23E-02	5.05E-01	1.60E-02	9.47E-01
**DURATION ABOVE 10 LUX AFTER SUNSET**	5.03E-01	2.42E-01	4.37E-01	7.69E-01	8.54E-01	5.58E-01	5.55E-01	3.99E-01	7.18E-01
**M10 MID-TIME**	4.06E-01	9.79E-01	4.18E-01	6.41E-01	7.82E-01	2.80E-01	6.38E-02	9.60E-01	5.26E-01
**L5 MID-TIME**	6.96E-01	1.22E-02	3.26E-01	5.56E-01	9.80E-02	7.42E-01	3.09E-01	8.12E-02	2.18E-01
**LAST TIME ABOVE 1 LUX**	1.14E-02	2.05E-02	5.55E-01	5.39E-01	1.75E-01	5.03E-01	2.94E-01	8.65E-02	3.25E-01
**IS**	8.48E-01	1.56E-02	2.39E-02	3.23E-01	1.06E-01	4.69E-01	7.16E-01	3.99E-02	2.90E-01
**IV**	7.05E-01	1.25E-02	8.69E-02	9.32E-01	2.10E-02	4.77E-01	7.61E-01	7.83E-04	5.26E-01
									**sample sizes**
**M10**	56.000	56.000	56.000	56.000	56.000	56.000	56.000	56.000	56.000
**L5**	56.000	56.000	56.000	56.000	56.000	56.000	56.000	56.000	56.000
**DURATION ABOVE 250 LUX**	56.000	56.000	56.000	56.000	56.000	56.000	56.000	56.000	56.000
**DURATION ABOVE 10 LUX AFTER SUNSET**	56.000	56.000	56.000	56.000	56.000	56.000	56.000	56.000	56.000
**M10 MID-TIME**	56.000	56.000	56.000	56.000	56.000	56.000	56.000	56.000	56.000
**L5 MID-TIME**	56.000	56.000	56.000	56.000	56.000	56.000	56.000	56.000	56.000
**LAST TIME ABOVE 1 LUX**	56.000	56.000	56.000	56.000	56.000	56.000	56.000	56.000	56.000
**IS**	56.000	56.000	56.000	56.000	56.000	56.000	56.000	56.000	56.000
**IV**	56.000	56.000	56.000	56.000	56.000	56.000	56.000	56.000	56.000

Supplementary information

Table S8: Correlations between in-lab light sensitivity measures and average cognitive performance (Pearson correlation r, p value, sample size).

	KSS	PVT MEDIANRT	PVT ACCURACY	NB3 MEDIANRT	NB3 FPR	NB3 FNR	VS IES	VS FPR	VS FNR
									**Pearson correlation r**
**BRIGHTNESS .75 THRESHOLD**	-0.088	0.362	-0.101	-0.256	0.272	0.390	0.145	0.140	0.222
**BRIGHTNESS PREFERENCE**	0.305	-0.326	-0.086	-0.244	-0.220	-0.086	-0.235	-0.131	-0.260
**PIPR RESPONSE**	-0.058	-0.086	0.325	0.324	-0.074	-0.167	0.090	-0.210	-0.241
**PIPR RESPONSE (NORM)**	-0.117	-0.134	0.292	0.326	-0.105	-0.184	0.100	-0.222	-0.240
**INITIAL CONSTRICTION**	-0.377	0.056	-0.154	-0.076	0.196	-0.068	0.005	0.434	0.248
**SUBJECTIVE BRIGHTNESS**	0.303	-0.186	0.324	-0.023	-0.365	-0.292	-0.180	-0.374	-0.415
									**p-values**
**BRIGHTNESS .75 THRESHOLD**	6.70E-01	6.89E-02	6.25E-01	2.07E-01	1.80E-01	4.89E-02	4.81E-01	4.96E-01	2.76E-01
**BRIGHTNESS PREFERENCE**	5.56E-02	4.02E-02	5.97E-01	1.29E-01	1.72E-01	5.99E-01	1.45E-01	4.21E-01	1.06E-01
**PIPR RESPONSE**	7.17E-01	5.92E-01	3.82E-02	3.87E-02	6.44E-01	2.98E-01	5.74E-01	1.87E-01	1.29E-01
**PIPR RESPONSE (NORM)**	4.65E-01	4.04E-01	6.41E-02	3.77E-02	5.13E-01	2.50E-01	5.32E-01	1.64E-01	1.30E-01
**INITIAL CONSTRICTION**	1.50E-02	7.30E-01	3.36E-01	6.38E-01	2.20E-01	6.74E-01	9.75E-01	4.56E-03	1.18E-01
**SUBJECTIVE BRIGHTNESS**	5.37E-02	2.45E-01	3.87E-02	8.87E-01	1.89E-02	6.39E-02	2.59E-01	1.61E-02	6.92E-03
									**sample sizes**
**BRIGHTNESS .75 THRESHOLD**	26.000	26.000	26.000	26.000	26.000	26.000	26.000	26.000	26.000
**BRIGHTNESS PREFERENCE**	40.000	40.000	40.000	40.000	40.000	40.000	40.000	40.000	40.000
**PIPR RESPONSE**	41.000	41.000	41.000	41.000	41.000	41.000	41.000	41.000	41.000
**PIPR RESPONSE (NORM)**	41.000	41.000	41.000	41.000	41.000	41.000	41.000	41.000	41.000
**INITIAL CONSTRICTION**	41.000	41.000	41.000	41.000	41.000	41.000	41.000	41.000	41.000
**SUBJECTIVE BRIGHTNESS**	41.000	41.000	41.000	41.000	41.000	41.000	41.000	41.000	41.000
