# Supplementary material for: Relationships between light exposure and aspects of cognitive function in everyday life
Source: Commun Psychol. 2025 Dec 16;4:5. doi: 10.1038/s44271-025-00373-9 (PMC12789024; doi:10.1038/s44271-025-00373-9)
Supplement: Supplementary file 3 — Reporting Summary [file 44271_2025_373_MOESM3_ESM.pdf]

nature portfolio

Corresponding author(s): Altug Didikoglu, Robert J Lucas

Last updated by author(s): Nov 3, 2025

Reporting Summary

Nature Portfolio wishes to improve the reproducibility of the work that we publish. This form provides structure for consistency and transparency in reporting. For further information on Nature Portfolio policies, see our Editorial Policies and the Editorial Policy Checklist.

Statistics

For all statistical analyses, confirm that the following items are present in the figure legend, table legend, main text, or Methods section.

n/a	Confirmed	
☐	☒	The exact sample size (*n*) for each experimental group/condition, given as a discrete number and unit of measurement
☐	☒	A statement on whether measurements were taken from distinct samples or whether the same sample was measured repeatedly
☐	☒	The statistical test(s) used AND whether they are one- or two-sided<br>*Only common tests should be described solely by name; describe more complex techniques in the Methods section.*
☐	☒	A description of all covariates tested
☐	☒	A description of any assumptions or corrections, such as tests of normality and adjustment for multiple comparisons
☐	☒	A full description of the statistical parameters including central tendency (e.g. means) or other basic estimates (e.g. regression coefficient) AND variation (e.g. standard deviation) or associated estimates of uncertainty (e.g. confidence intervals)
☐	☒	For null hypothesis testing, the test statistic (e.g. *F*, *t*, *r*) with confidence intervals, effect sizes, degrees of freedom and *P* value noted<br>*Give P values as exact values whenever suitable.*
☒	☐	For Bayesian analysis, information on the choice of priors and Markov chain Monte Carlo settings
☐	☒	For hierarchical and complex designs, identification of the appropriate level for tests and full reporting of outcomes
☐	☒	Estimates of effect sizes (e.g. Cohen's *d*, Pearson's *r*), indicating how they were calculated

Our web collection on statistics for biologists contains articles on many of the points above.

Software and code

Policy information about availability of computer code

Data collection	Software and hardware designs of the wearable light dosimeter are available in a repository (https://github.com/Non-Invasive-Bioelectronics-Lab/Wearable_Light_Sensor_Public). Behavioural and cogintive data were collected using Brightertime mobile app (https://doi.org:10.3390/ clockssleep4040045).
Data analysis	All data processing and analyses were conducted using R version 4.4.1 (2024) and MATLAB version R2023b, with data visualization performed in GraphPad Prism version 10.3.0.

For manuscripts utilizing custom algorithms or software that are central to the research but not yet described in published literature, software must be made available to editors and reviewers. We strongly encourage code deposition in a community repository (e.g. GitHub). See the Nature Portfolio guidelines for submitting code & software for further information.

Data

Policy information about availability of data

All manuscripts must include a data availability statement. This statement should provide the following information, where applicable:

- Accession codes, unique identifiers, or web links for publicly available datasets
- A description of any restrictions on data availability
- For clinical datasets or third party data, please ensure that the statement adheres to our policy

Anonymized data of light exposure and cognitive tasks created for the study will be available in a Figshare repository.

Research involving human participants, their data, or biological material

Policy information about studies with human participants or human data. See also policy information about sex, gender (identity/presentation), and sexual orientation and race, ethnicity and racism.

Reporting on sex and gender	The sample consisted of 29 males and 29 females. Sex was used as a covariate in the analysis. Sex and gender were determined based on information provided by the participants.
Reporting on race, ethnicity, or other socially relevant groupings	The study was conducted between July 2022 and August 2023 in Manchester, UK. Participants were residents of the UK but no race or ethnicity information was collected in the study.
Population characteristics	Participants were eligible for inclusion if they were at least 18 years old, employed either full-time or part-time, had no history of intercontinental travel in the preceding two weeks, and had not been diagnosed with a sleep disorder. The study aimed to capture a real-world population; therefore, no specific health-related exclusion criteria were applied. Age distribution was as follows: 14 participants were <25 years old, 23 were between 25 and 30, 13 were between 30 and 35, and the remaining participants were over 35 years old. The majority (n = 55) held a higher education degree, and 33 were employed full-time. Four participants occasionally engaged in shift work, with eight days reported as shift work during the study; however, none involved night shifts that significantly alter night sleep patterns. Regarding health characteristics, 46 participants were non-smokers, two were colorblind, three had an ADHD diagnosis, and 11 reported anxiety or depression.
Recruitment	Recruitment was conducted using social media posts, posters in University of Manchester buildings, announcements on the University of Manchester research study volunteering web page, and word of mouth.
Ethics oversight	The University of Manchester

Note that full information on the approval of the study protocol must also be provided in the manuscript.

Field-specific reporting

Please select the one below that is the best fit for your research. If you are not sure, read the appropriate sections before making your selection.

☐ Life sciences ☒ Behavioural & social sciences ☐ Ecological, evolutionary & environmental sciences

For a reference copy of the document with all sections, see nature.com/documents/nr-reporting-summary-flat.pdf

Behavioural & social sciences study design

All studies must disclose on these points even when the disclosure is negative.

Study description	An observational study conducted in naturalistic settings, with minimal exclusion criteria and minimal interference with participants' everyday life. Quantitative data were collected on light exposure, sleep, cognition, and daily behavior.
Research sample	The sample includes residents of Manchester, UK, primarily young adults, with an approximately equal distribution of males and females. The findings may not be directly generalizable to older adults, individuals with physical or mental health conditions, or those in occupations such as night shift work.
Sampling strategy	To ensure sufficient statistical power, a minimum sample size of 50 participants was targeted. This was based on published power analysis recommendations for similar research on light exposure in real-world settings (https://doi.org/10.1371/journal.pone.0308768). Convenience sampling was used. Recruitment was conducted via social media posts, posters in University of Manchester buildings, announcements on the University of Manchester research study volunteering webpage, and word of mouth.
Data collection	Light exposure data were collected using a wrist-worn wearable light sensor (Spectrawear), with data stored in the device's internal memory. At the end of the study, the data were retrieved by the researcher and anonymised using participant study IDs. Cognitive, sleep, behavioural, sociodemographic, and survey data were collected via a mobile application (Brightertime), which participants accessed using their personal smartphones. These data were directly anonymised and securely stored in the University of Manchester's research cloud storage system.
Timing	The study was conducted between July 2022 and August 2023.
Data exclusions	Two participants were excluded from the final analyses due to study design noncompliance and insufficient data provision.
Non-participation	Two participants dropped out after starting the experiment. One withdrew due to a device malfunction and was unwilling to continue with a replacement device. The other participant felt the study protocol did not fit with their everyday life and believed they would not be able to provide sufficient data.
Randomization	The study was observational and correlational in design, with no group allocation or randomisation.

Reporting for specific materials, systems and methods

We require information from authors about some types of materials, experimental systems and methods used in many studies. Here, indicate whether each material, system or method listed is relevant to your study. If you are not sure if a list item applies to your research, read the appropriate section before selecting a response.

Materials & experimental systems

n/a	Involved in the study
☒	☐ Antibodies
☒	☐ Eukaryotic cell lines
☒	☐ Palaeontology and archaeology
☒	☐ Animals and other organisms
☒	☐ Clinical data
☒	☐ Dual use research of concern
☒	☐ Plants

Methods

n/a	Involved in the study
☒	☐ ChIP-seq
☒	☐ Flow cytometry
☒	☐ MRI-based neuroimaging

Plants

Seed stocks

Report on the source of all seed stocks or other plant material used. If applicable, state the seed stock centre and catalogue number. If plant specimens were collected from the field, describe the collection location, date and sampling procedures.

Novel plant genotypes

Describe the methods by which all novel plant genotypes were produced. This includes those generated by transgenic approaches, gene editing, chemical/radiation-based mutagenesis and hybridization. For transgenic lines, describe the transformation method, the number of independent lines analyzed and the generation upon which experiments were performed. For gene-edited lines, describe the editor used, the endogenous sequence targeted for editing, the targeting guide RNA sequence (if applicable) and how the editor was applied.

Authentication

Describe any authentication procedures for each seed stock used or novel genotype generated. Describe any experiments used to assess the effect of a mutation and, where applicable, how potential secondary effects (e.g. second site T-DNA insertions, mosiacism, off-target gene editing) were examined.
